# YTHDF1 impacts cardiomyocyte differentiation by regulating the *TET2* mRNA

**DOI:** 10.1371/journal.pone.0349040

**Published:** 2026-05-15

**Authors:** Guanlin Zheng, Banban Li, Maochuan Zheng, Xiuli Tian, Zhiyong Li, Hongwei Shi, Xiaoxiao Xu, Li Fan, Yajun Hu, Liyan Jing

**Affiliations:** 1 Postdoctoral Research Workstation, The Affiliated Taian City Central Hospital of Qingdao University, Taian city, Shandong Province, P.R. China; 2 Department of Midwifery, Taishan Vocational College of Nursing, Taian city, Shandong Province, P.R. China; 3 Department of Critical Care Medicine, The Second Affiliated Hospital of Shandong First Medical University, Taian city, Shandong Province, P.R. ‌‌‌China‌‌; 4 Department of Pathology, Faculty of Medical Imaging, Naval Medical University, Shanghai, P.R. China; University of Toronto, CANADA

## Abstract

Cardiac diseases frequently arise from compromised cardiomyocyte differentiation and function. Although studies have demonstrated that loss of the m^6^A modification reader YTHDF1 impairs cardiomyocyte differentiation, the specific mRNA transcripts it directly regulates remain to be identified. Comprehensive analysis of public databases was conducted to examine the correlation between YTHDF1 expression and cardiac differentiation processes as well as specific cardiac pathology. Stable YTHDF1-knockdown cell lines were generated in the rat H9C2 cardiomyoblasts. After retinoic acid (RA) induction, cardiomyocyte differentiation was assessed. RNA immunoprecipitation sequencing (RIP-seq) was performed in H9C2 cells, and the resulting data were integrated with known cardiomyocyte differentiation regulators to identify direct YTHDF1 mRNA targets. Here we found that YTHDF1 expression increases progressively during cardiomyocyte differentiation but gradually declines upon cellular/organ maturation in mice. Human left ventricle (LV) exhibited higher YTHDF1 expression than right ventricle (RV), while LV from dilated cardiomyopathy (DCM) patients showed modestly reduced YTHDF1 levels compared to healthy controls. In differentiating H9C2 cardiomyoblasts, YTHDF1 expression progressively increased. YTHDF1 knockdown impaired differentiation, reducing maturation markers cTnT/cTnI, which was aligned with RNA-seq analysis. RIP-seq identified significant *TET2* mRNA enrichment in YTHDF1 complexes. YTHDF1 knockdown selectively reduced TET2 protein level without affecting its mRNA level, while YTHDF1 overexpression enhanced TET2 translation. Additionally, we identified a novel rat *tet2* variant. Complementation with this variant in YTHDF1-knockdown H9C2 cells rescued the differentiation defect. Collectively, YTHDF1 promotes cardiomyocyte differentiation by regulating *TET2* mRNA during cardiac development.

## Introduction

Defects in cardiomyocyte differentiation directly contribute to various congenital cardiac defects and cardiomyopathies [1,2]. Despite extensive research, the comprehensive molecular mechanisms and signaling networks orchestrating cardiomyocyte differentiation remain incompletely characterized.

Epigenetic modification represents a crucial natural mechanism for regulating gene expression through various modifications at the DNA, histone, and RNA levels without altering the genomic sequence [3,4]. Among mRNA modifications in mammalian cells, N6-methyladenosine (m^6^A) represents the most abundant type [5]. Previous studies have demonstrated that during *in vitro* differentiation of human embryonic stem cells (hESCs) into cardiomyocytes, the m^6^A methylation level of mRNA increases significantly, particularly during the late differentiation stage when mesodermal progenitors transition to committed cardiomyocytes [6]. Additionally, cardiovascular-specific knockout of methyltransferase-like 3 (METTL3), a m^6^A writer, leads to insufficient m^6^A RNA methylation, resulting in congenital cardiac defects and postnatal lethality in mice [7]. Therefore, the regulatory role of mRNA m^6^A modification in key genes during cardiomyocyte differentiation warrants further investigation.

The YT521-B homology (YTH) domain-containing family proteins (YTHDFs) represent a class of evolutionarily conserved mRNA m^6^A readers that include YTHDF1, YTHDF2, and YTHDF3, which can mediate mRNA translation or degradation [8]. All members contain a C-terminal YTH domain that binds mRNA and specifically recognizes m^6^A modification sites [9], while their N-terminal low-complexity regions (LCRs) exhibit significant divergence, leading to distinct RNA-binding protein interactions—a key structural feature that determines their effects on mRNA translation or degradation [8]. YTHDF1 regulates mRNA translation by recognizing m^6^A-modified mRNAs and delivering them to specific effectors for protein synthesis [8]. Some studies in cardiovascular diseases have revealed that YTHDF1 exerts cardioprotective effects during myocardial injury by mediating the translation of YAP [10,11], CAV1 [12], FTH1 [13] and RBM4 [14] mRNAs. However, it may also exacerbate apoptosis or cardiac fibrosis through upregulating the expression of FOXO3a [15], PIEZO2 [16], MeCP2 [17], and AXL [18] proteins. Despite these established discoveries, investigations into YTHDF1’s function in cardiomyocyte differentiation remain scarce. To our knowledge, only one *in vitro* study indicates that YTHDF1 depletion severely impairs the differentiation of embryonic stem cells (ESCs) into cardiomyocytes [19], although the precise target mRNAs regulated by YTHDF1 in this circumstance await elucidation.

Ten-eleven translocation (TET) family proteins (TETs) are a crucial class of DNA demethylases. They utilize the conserved C-terminal dioxygenase domain to sequentially catalyze the oxidation of 5-methylcytosine (5mC) into 5-hydroxymethylcytosine (5hmC), 5-formylcytosine (5fC), and 5-carboxylcytosine (5caC). Subsequently, 5caC recognized by DNA glycosylases and replaced with unmodified cytosine through base excision repair (BER), thereby achieving active DNA demethylation [20]. *In vitro* studies revealed that knockout of TETs in hESCs leads to hyperactivation of Wnt signaling and abolishes their differentiation into cardiomyocytes [21]. And knockdown of TET2 alone is sufficient to inhibit the differentiation of hESCs into cardiomyocytes [22,23]. Furthermore, cardiac-specific knockout of TET2/3 in mice results in left ventricular non-compaction cardiomyopathy with embryonic lethality [24]. These findings collectively demonstrate the critical role of the TET family, especially TET2, in regulating cardiomyocyte differentiation.

In this study, we further investigated the critical role of YTHDF1 in regulating cardiomyocyte differentiation. YTHDF1 is upregulated during the differentiation of rat cardiac myoblast H9C2 cell line. Knockdown of YTHDF1 in H9C2 significantly inhibits their differentiation into cardiomyocytes. RNA immunoprecipitation (RIP) analysis revealed that *TET2* mRNA is one of the key targets of YTHDF1. Upon YTHDF1 knockdown, the transcriptional level of TET2 in H9C2 remained unchanged, while its protein level decreased significantly. Notably, a novel rat *tet2* variant successfully rescued the differentiation defect in YTHDF1-knockdown cells. These findings demonstrate that YTHDF1 binds *TET2* mRNA and regulates its translation, thereby driving the sequential expression of key genes essential for cardiomyocyte differentiation.

## Method

### 1. Cell culture and induced differentiation

H9C2 cells were cultured in high-glucose DMEM supplemented with 10% fetal bovine serum (FBS), 1% penicillin/streptomycin and maintained in a 37°C, 5% CO₂ incubator (Bluepard, China), with the medium replaced daily. When cell density reached 90% confluence, passaging was performed promptly. To establish stable YTHDF1-overexpressing or knockdown cell lines, cells were infected with lentivirus carrying rat-YTHDF1-overexpressing or -shRNA plasmids (OBiO Technology, China) for 48 hours, followed by puromycin selection (5 μg/mL) for one week. The shRNA target sequences are listed in Supplementary Table 1 (S1 Table). Throughout this article, cells transfected with the KD2 sequence are referred to as sh-YTHDF1 or KD cells.

For differentiation induction, cells were grown to 100% confluence and then switched to differentiation medium containing 1% fetal bovine serum (FBS) and 1 μM retinoic acid (RA). The induction lasted for 5 days, with the medium refreshed daily. For transient plasmid transfection, cells were transfected with plasmids using polyethylenimine (PEI) (Yeasen, China) one day prior to differentiation induction.

### 2. Cell proliferation assay

Cells were seeded in 96-well plates at a density of 1,000 cells per well. After cell attachment (about 5 hours) and subsequently every 24 hours, CCK-8 reagent was added, followed by incubation at 37°C for 1 hour. The absorbance at 450 nm was measured using a microplate reader (Thermo Scientific, U.S.). To assess proliferation under hypoxia-reoxygenation conditions, cells were subjected to 24-hour hypoxia/nutrient deprivation (glucose-free medium under hypoxic conditions) after attachment, followed by refeeding with high-glucose DMEM under normal culture conditions for five days.

### 3. Bulk RNA-seq and bioinformatics analysis

Total RNA was isolated from cell samples using TRIzol reagent (Accurate Biology, China) according to the manufacturer’s protocol. RNA samples were subsequently submitted at a commercial facility (OBiO Technology, China) for next-generation sequencing (NGS) and bioinformatic analysis, including differential gene expression analysis and functional enrichment analysis. The processed RNA-Seq FPKM data were provided as Supplementary Table 4. R studio software and GraphPad Prism software were employed for data visualization.

### 4. RNA immunoprecipitation sequencing (RIP-seq)

RIP was conducted by using RNA Immunoprecipitation kit (BersinBio, China). Briefly, 2 × 10^7^ H9C2 cells were harvest freshly, and lysis on ice for 30 min. After DNA digestion, cell lysate was divided into three parts, 0.8 mL (for IP sample), 0.8 mL (for IgG sample), and 0.1 mL (as Input sample). Anti-YTHDF1 and Anti-IgG was added into IP sample and IgG sample respectively followed by overnight incubation at 4℃. Equal balanced protein A/G magnetic beads were then added into IP sample and IgG sample for conjugation. After 2 hours, beads were washed and RNA in three samples were all extracted for RIP sequencing (OBiO Technology, China). The data were provided as Supplementary Table 5.

### 5. Western blot (WB) and dot blot

For Western blot, cells were washed three times with phosphate buffered saline (PBS) and lysed in ice-cold RIPA buffer supplemented with protease inhibitor cocktail for 20 min on ice. The lysates were collected using a cell scraper and centrifuged at 12,000 rpm for 20 min. The supernatant was collected for protein quantification using BCA assay. Equal amounts of protein were mixed with denaturing and loading buffer, denatured at 100°C for 10 min, and immediately cooled on ice. Proteins were separated by SDS-PAGE and transferred to PVDF membranes. After blocking, membranes were incubated with primary antibodies at 4°C overnight, followed by incubation with appropriate HRP-conjugated secondary antibodies at room temperature (RT). Protein bands were visualized using ECL substrate kit and imaged with a chemiluminescence detector (Cytiva, Sweden).

For dot blot, genomic DNA was extracted from fresh cells using a DNA extraction kit. Equal amounts of DNA were denatured in DNA denaturation buffer at 100°C for 5 minutes and immediately chilled on ice. Nitrocellulose membranes were pre-wetted with 2 × saline-sodium citrate (SSC) buffer and air-dried. DNA samples (10 μL) were carefully spotted onto membranes, which were then cross-linked at 80°C. After blocking with 5% non-fat milk for 1 hour, the membranes were processed for antibody incubation following the same procedure as Western blot.

All antibodies used in this study are listed in Supplementary Table 3 (S3 Table).

### 6. Quantitative polymerase chain reaction (Q-PCR)

Total RNA was extracted from cells using TRIzol reagent following the manufacturer’s protocol. RNA was reverse-transcribed into cDNA using a reverse transcription kit. Q-PCR was performed using a SYBR Green-based Taq polymerase master mix (Accurate Biology, China) on a real-time PCR system (Bioer, China). Data were analyzed by △△Ct. All primer sequences were listed in Supplementary Table 2 (S2 Table).

### 7. Data processing and statistical analysis

All experimental data were analyzed and visualized using GraphPad Prism software. Statistical significance was assessed using Student’s t-test for two-group comparisons and one-way or two-way ANOVA followed by Tukey’s or Dunnett’s multiple comparisons test for multi-group analyses. Details of the statistical methods are provided in the figure legend. The significance level was marked as ‘*’ (p < 0.05), ‘**’ (p < 0.01), ‘***’ (p < 0.001) and ‘****’ (p < 0.0001).

## Results

### 1. YTHDF1 expression level in heart show correlation with cardiomyocyte differentiation

**Fig 1:**
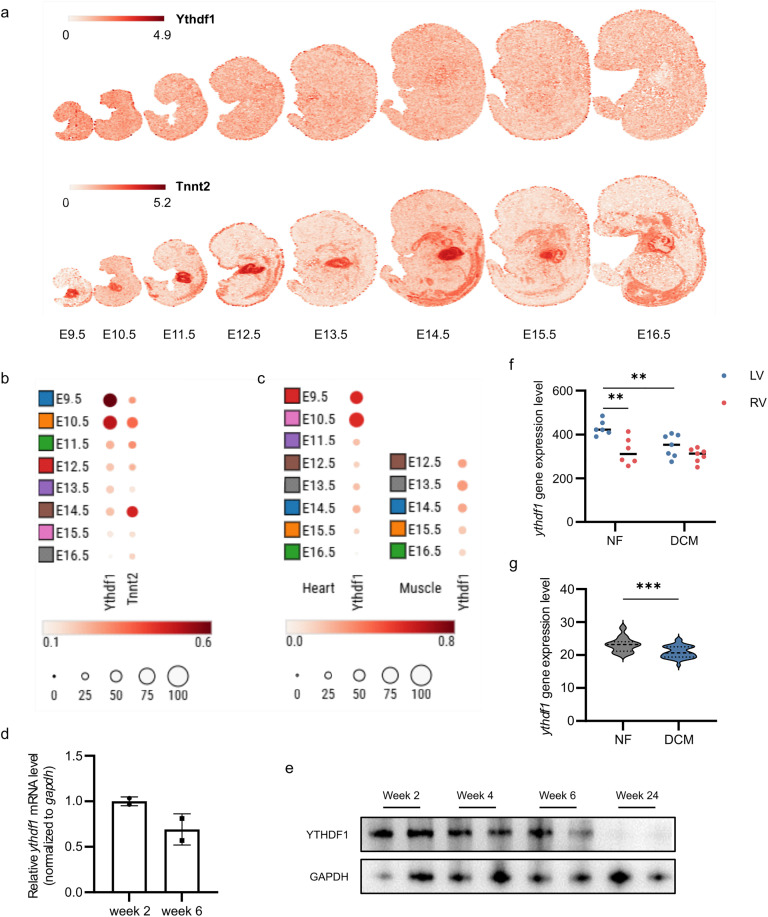
YTHDF1 expression during cardiac development and in dilated cardiomyopathy. **a.** Expression patterns of YTHDF1 (up) and cTnT (cardiac troponin T, encoded by *tnnt2*) (down, a marker of heart) in mouse embryo from embryonic day 9.5 (E9.5) to embryonic day 16.5 (E16.5). **b, c.** Bubble plots of the expression of YTHDF1 and cTnT in whole mouse embryo (**b**) and the expression of YTHDF1 in mouse embryonic heart and muscle from E9.5 to E16.5 (**c**). Size, the percentage of expressed cells; Color, the mean expression level. **d.** mRNA level of YTHDF1 in mouse heart tissues at the age of 2 weeks and 6 weeks (n = 2). **e.** Protein level of YTHDF1 in mouse heart tissues at the age of 2 weeks, 4 weeks, 6 weeks and 24 weeks. Each stage has two biological replicates. **f.** YTHDF1 gene expression level in the hearts of none-failing people (NF) and dilated cardiomyopathy (DCM) from GSE29819. LV, left ventricle; RV, right ventricle. Unpaired two-tailed t test and paired two-tailed t test were used (n = 6/ n = 7). **g.** YTHDF1 gene expression level in the left ventricle of none-failing people (NF) and dilated cardiomyopathy (DCM) from GSE116250. Unpaired two-tailed t test was used (n = 14/ n = 37)‌‌.

The heart is the first organ to form and become functional during embryonic development. In mice, the heart typically develops into a four-chambered structure between E7.5 and E12.5 [25], with E11.5 to E14.5 being a critical phase for cardiomyocyte proliferation and differentiation to thicken the ventricular walls [26]. From analysis of the mouse organogenesis spatiotemporal transcriptomic atlas (MOSTA) [27], it revealed that compared to later stages, YTHDF1 exhibits peak expression levels and the broadest tissue distribution throughout the entire embryo at E9.5-E10.5, followed by a rapid decline (Fig 1a, 1b). Consistently in cardiac tissue, expression level of YTHDF1 significantly decreases after E10.5 (Fig 1c). Notably, a slight upregulation is observed in both heart and muscle tissues from E12.5 to E14.5 (Fig 1c). Meanwhile, postnatal murine hearts also exhibit a progressive decline in YTHDF1 protein expression levels while transcription level has no significant change (Figs 1d, 1e, S1a), suggesting the potential role of YTHDF1 in cardiac development. Analysis of GEO datasets (GSE29819) revealed that in non-failing (NF) donor hearts, YTHDF1 expression levels were significantly higher in the left ventricle (LV) compared to the right ventricle (RV). Notably, patients with dilated cardiomyopathy (DCM) exhibited a marked downregulation of YTHDF1 expression in the left ventricle (Fig 1f). Similar result was found in the data of GSE116250 (Fig 1g). Based on the impaired cardiomyocyte differentiation contributing to compromised contractility in DCM, these results strongly suggest that YTHDF1 may participate in the molecular regulation of cardiomyocyte differentiation.

### 2. Knockdown of YTHDF1 inhibits rat cardiomyocytes differentiation *in vitro*

**Fig 2:**
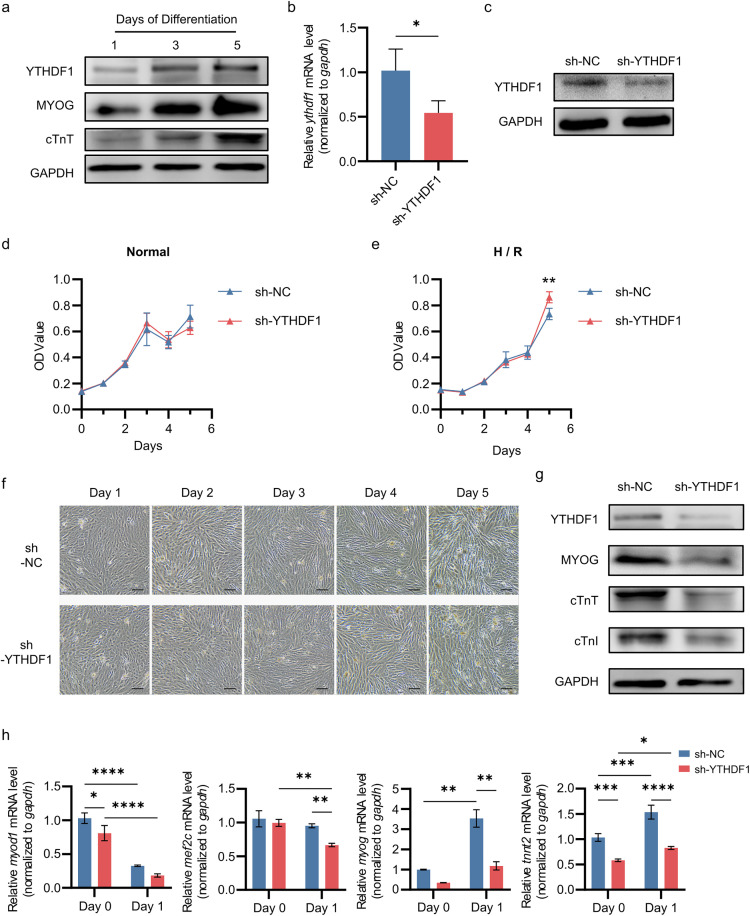
YTHDF1 knockdown impairs H9C2 cells differentiation toward cardiomyocytes. **a.** Protein levels of YTHDF1 and several differentiation markers during H9C2 cardiomyocyte differentiation. **b, c.** Relative mRNA level (**b**) and protein level (**c**) of YTHDF1 in YTHDF1-knockdown H9C2 cell lines. Unpaired two-tailed t test was used (n = 3). **d, e.** Proliferation of H9C2 YTHDF1-knockdown cells under normoxia (Normal) (**d**) and hypoxia/ reoxygenation (H/ R) conditions (**e**). Two-way ANOVA was used (n ≥ 3). **f.** Morphological changes during differentiation in YTHDF1-knockdown cells versus control H9C2 cells. Lines, 100 μm. **g.** Protein levels of marker genes in YTHDF1-knockdown and control H9C2 cell lines at the late-stage induced differentiation (Day 4). **h.** Transcript levels of cardiomyocyte differentiation markers in YTHDF1-knockdown and control H9C2 cell lines before (Day 0) and at early stage of induced differentiation (Day 1). Two-way ANOVA was used (n = 3).

The H9C2 cell line, derived from rat embryonic cardiac tissue, is widely used to study early cardiac developmental processes [28]. In our retinoic acid-induced H9C2 cardiomyocyte differentiation model, we observed a gradual increase in the protein levels of MYOG, a key transcription factor for myocyte differentiation; alongside elevated levels of cardiac troponin T (cTnT), a marker of cardiomyocyte maturation (Fig 2a). Consistently, YTHDF1 protein expression was also significantly upregulated during this process (Fig 2a). To investigate the functional role of YTHDF1 in cardiomyocyte proliferation and differentiation, we generated YTHDF1-knockdown H9C2 cell lines using lentiviral vectors, with knockdown efficiency validated at both transcriptional and protein levels (Fig 2b, 2c). Under normoxic conditions, proliferation rates showed no significant difference between knockdown and control cells (Fig 2d), while hypoxia/reoxygenation (H/R) stress may moderately enhance YTHDF1-knockdown cells to proliferate (Fig 2e).

We further investigated the role of YTHDF1 in H9C2 cells differentiation toward cardiomyocytes. After 5 days of induction, while both groups displayed similar elongated cellular morphology, the protein level of cardiac troponin T (cTnT) and cardiac troponin I (cTnI) were significantly lower in YTHDF1-knockdown cells than controls on Day 4, suggesting the functional deficiency in induced YTHDF1-knockdown cells (Fig 2f, 2g). Then we analyzed the expression patterns of key regulatory factors during the early induction phase. MYOD1, the molecular determinant of myogenic progenitor cell fate commitment, exhibited a slight decrease in mRNA level upon YTHDF1 knockdown prior to induction (Fig 2h), although MYOD1 was sharply downregulated in both groups at the beginning of induction likely due to the primary involvement of MYOD1 in skeletal muscle cell differentiation but not cardiomyocytes (Fig 2h) [29^]^. Myogenin (MYOG), which primarily regulates myoblast fusion and myotube formation, showed rapid transcriptional upregulation at the initial stage of induction [30]. YTHDF1 knockdown significantly suppressed MYOG expression compared to the control group (Fig 2g, 2h). Similarly, MEF2c—a crucial regulator of myogenic differentiation and maturation—demonstrated lower transcriptional levels in YTHDF1-knockdown cells during early differentiation induction (Fig 2h) [31]. Additionally, YTHDF1 knockdown resulted in reduced transcriptional levels of *tnnt2* (encoding cTnT) in both Day 0 and Day1 compared to control cells (Fig 2h). These results demonstrate that YTHDF1 knockdown significantly impairs the differentiation capacity of H9C2 cells toward cardiomyocytes.

**Fig 3:**
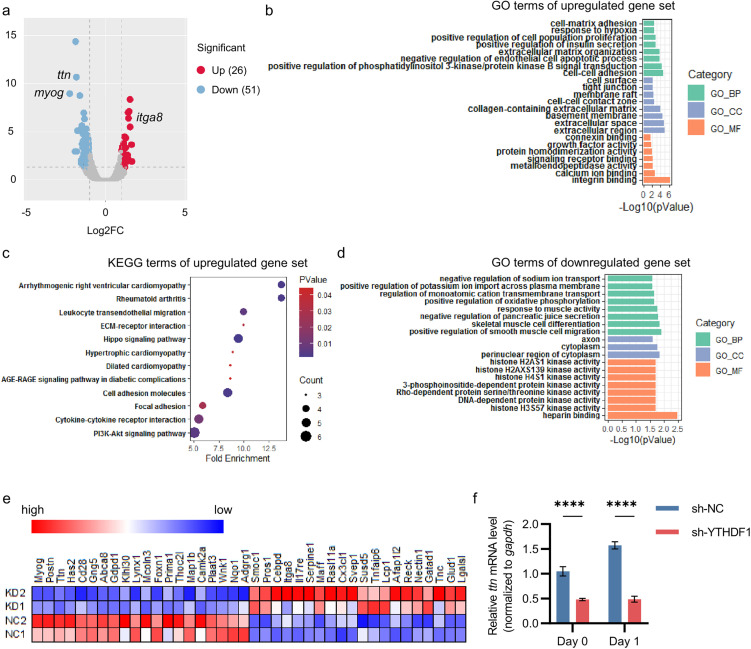
Knockdown of YTHDF1 perturbs the expression of genes and pathways associated with myocyte differentiation and certain cardiac diseases. **a.** Volcano plot of significantly differentially expressed genes (p < 0.05, |Log2FC| > 1, FDR < 0.05) between YTHDF1-knockdown and control H9C2 cell lines. **b-d,** Enrichment analysis of up-regulated (**b**, **d**) and down-regulated (**d**) genes. GO, Gene Ontology; KEGG, Kyoto Encyclopedia of Genes and Genomes. **e.** Hot plot for the normalized FPKM value of top 20 upregulated genes and top 20 downregulated genes. NC, sh-NC; KD, sh-YTHDF1. Both of them have independent replicates. **f.** Transcript levels of TTN in YTHDF1-knockdown and control H9C2 cell lines before (Day 0) and at early stage of induced differentiation (Day 1). Two-way ANOVA was used (n = 3).

RNA-seq analysis revealed that compared with the control group of H9C2, the YTHDF1 knockdown group showed 26 significantly upregulated genes, including *itga8*; and 51 significantly downregulated genes, including *myog* and *ttn* (encodes a large abundant protein of striated muscle) (Fig 3a). Enrichment analysis demonstrated that the upregulated genes were primarily enriched in cell adhesion-related pathways and several cardiac diseases (Fig 3b, 3c), while the downregulated genes were predominantly enriched in pathways associated with myocyte migration and differentiation (Fig 3d). Top differentially expressed genes were listed in hot plot (Fig 3e). And the expression changes of *myog* and *ttn* were validated by Q-PCR (Fig 2h, 3f). These results aligned with the phenotypic observation in cellular experiments where YTHDF1 knockdown led to impaired differentiation of H9C2 cardiomyocytes.

### 3. YTHDF1 binds to *TET2* mRNA and regulates it translation

**Fig 4:**
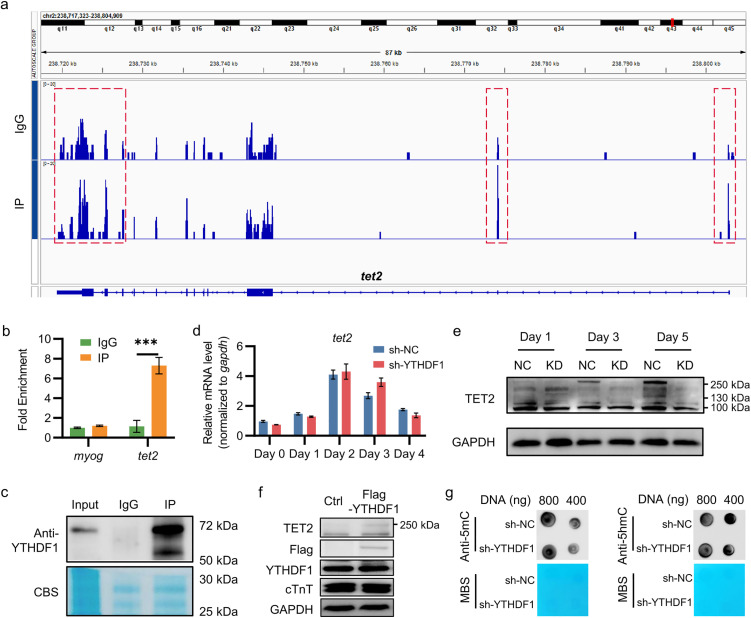
YTHDF1 directly regulates *TET2* mRNA translation. **a.** the integrative genomics viewer (IGV) plot of the differential enrichment peaks of *TET2* mRNA between the IgG group and the IP group by the RIP-seq. **b.** the quantification of *MYOG* and *TET2* mRNA levels in RIP samples by Q-PCR. Unpaired two-tailed t test was used (n = 3). **c.** Validation of the conjugation of YTHDF1 in RIP experiment by WB. CBS, Coomassie Blue Staining. **d, e.** the mRNA (**d**) and protein (**e**) levels of TET2 during H9C2 cells differentiation with or without YTHDF1 gene knockdown. NC, sh-NC; KD, sh-YTHDF1. Two-way ANOVA was used (n = 3). **f.** the relative protein levels of TET2, YTHDF1, cTnT in H9C2 cells differentiated at day 4 with or without YTHDF1 gene overexpression. **g.** the DNA 5mC and 5hmC modification level in H9C2 cells differentiated at day 5 with or without YTHDF1 gene knockdown. MBS, Methylene Blue Staining‌‌.

As an m^6^A reader, YTHDF1 canonically functions by binding and stabilizing mRNA translation. To identify YTHDF1 target mRNAs, we performed RNA Immunoprecipitation Sequencing (RIP-seq). Screening of the results revealed significant enrichment of *TET2* mRNA (Fig 4a), which encodes an important regulator during cardiomyocyte differentiation. This interaction was further validated by Q-PCR, confirming the direct association between *TET2* transcripts and YTHDF1 protein (Figs 4b, 4c). Notably, During H9C2 differentiation, TET2 was upregulated (Figs 4d, 4e, S1d, S1e). Knockdown of YTHDF1 markedly reduced TET2 protein level without significantly affecting its mRNA abundance (Figs 4d, 4e, S1b–S1e). When YTHDF1 had been overexpressed in H9C2, the upregulation of TET2 protein was observed, although the cTnT was not affected (Fig 4f). Despite a marked reduction in TET2 protein levels upon YTHDF1 knockdown during H9C2 differentiation, DNA 5mC and 5hmC levels remained comparable to control cells (Figs 4g, S1f, S1g). Sequencing data revealed that *tet3* transcript levels were substantially higher than *tet1* and *tet2* in H9C2 cells (S1h Fig). This unchanged methylation pattern can be attributed to the compensatory effect of high TET3 expression, which likely prevented global changes in DNA methylation despite the reduction in TET2 protein levels. Collectively, these results demonstrate that YTHDF1 binds to *TET2* mRNA and regulates its translation in H9C2 cells.

### 4. TET2 complementation rescues cardiomyocyte differentiation defects caused by YTHDF1 knockdown

**Fig 5:**
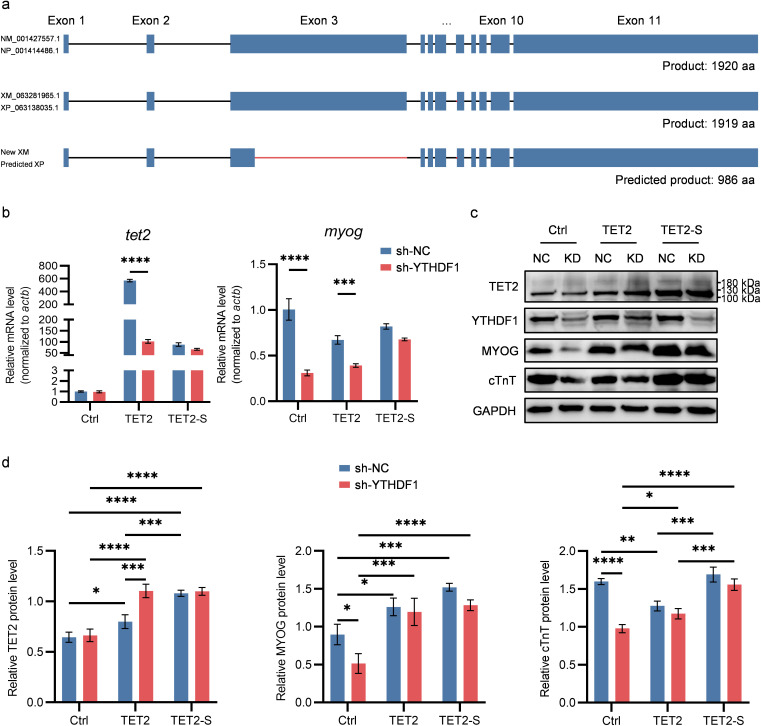
TET2 complementation rescues cardiomyocyte differentiation defects caused by YTHDF1 knockdown. **a.** Schematic of the three *tet2* mRNA variants. Blue rectangles represent exons, dark lines denote common introns, and red lines indicate exon regions absent in the canonical reference (NM_001427557.1). **b.** *TET2* and *MYOG* mRNA levels at day 5 of differentiation in YTHDF1-knockdown and control H9C2 cells with transient *tet2* variant complementation. Two-way ANOVA was used (n = 3). **c.** TET2, MYOG, and cTnT protein levels at day 5 of differentiation in YTHDF1-knockdown and control H9C2 cells with transient *tet2* variant complementation. NC, sh-NC; KD, sh-YTHDF1; Ctrl, control plasmid; TET2, canonical *tet2* isoform plasmid; TET2-S, new *tet2* isoform (S, short) plasmid. **d.** Quantification of TET2 (~100 kDa), MYOG, and cTnT protein levels from panel c (n = 3).

To investigate whether TET2 mediates YTHDF1’s regulatory role in H9C2 cardiomyocyte differentiation, we performed TET2 complementation in YTHDF1-knockdown cells. First, we constructed a CMV-driven overexpression plasmid encoding the full-length TET2 protein (1920 aa, ~ 212 kDa; Fig 5a) based on the rat *TET2* mRNA sequence (NM_001427557.1). Second, we prepared a cDNA library from H9C2 total RNA and amplified the *tet2* coding sequence by PCR. During this process, we unexpectedly identified a novel *tet2* transcript variant (designated New XM). Sequence alignment revealed that New XM differs from NM_001427557.1 through alternative splicing in exons 3 and 7, while sharing the same spliced sequence in exon 7 with the predicted variant XM_063281965.1 (Figs 5a, S2a). Specifically, the New XM is generated by the use of an alternative 5’ splice site within exon 3, which truncates the exon and results in the loss of 933 amino acids. The New XM also harbors a downstream-shifted splice site in exon 7 (S2a Fig), resulting in deletion of one amino acid in the region corresponding to exon 7 of the canonical isoform. Primers flanking the alternative splicing region were designed, and PCR amplification yielded a prominent fragment of approximately 1,000 bp, consistent with the predicted amplicon size of the coding sequence (CDS) for New XM (S2b Fig). Importantly, the level of *TET2* mRNA with relatively complete retention of exon 3 increased significantly upon differentiation, while YTHDF1 knockdown markedly attenuated this effect (S2c Fig). This finding is consistent with the marked reduction of the high-molecular-weight TET2 band (~250 kDa) in YTHDF1-knockdown cells compared to controls during the induction of cardiac differentiation (Fig 4e). The alternative splicing in the new variant is predicted to preserve its methylcytosine dioxygenase domain—a region encoded from the latter segment of exon 7 to the first half of exon 11 according to the Uniprot database. Analysis of the splice-junction sequence at exon 3 confirmed that it follows the canonical spliceosome recognition motif and shows conservation at corresponding nucleotide positions in human and mouse *tet2* (S2a, S2d Fig). The New XM variant is predicted to encode a 986-aa protein (~110 kDa). Transfection of this variant into 293T cells produced a markedly enhanced band at approximately 100 kDa (S2e Fig). Moreover, deepSRAMP prediction suggested that alternative splicing at exon 7 could potentially affect m⁶A modification for motif AAACT (S2f Fig). Amino acid sequence comparisons among the three variants are shown in S3 Fig.

Upon induction of differentiation, we transiently expressed two *TET2* mRNA variants in YTHDF1-knockdown and control H9C2 cells. By day 5, the new *tet2* variant more effectively restored MYOG expression and cTnT levels in YTHDF1-deficient cells (Fig 5b–5d), thereby rescuing the differentiation defect. Similarly, in sh-1 YTHDF1-knockdown cells, the new variant remained more effective in promoting MYOG transcription, although *tet2* transcript levels were significantly higher in cells transfected with the canonical *tet2* variant (NM_001427557.1) than in those transfected with the new *tet2* variant (S2g Fig). Collectively, these findings demonstrate that YTHDF1 promotes H9C2 cardiomyocyte differentiation by enhancing TET2 expression.

## Discussion

Wang S et al. [19] demonstrated that YTHDF1 promotes the differentiation of mouse embryonic stem cells into cardiomyocytes in vitro, although its direct mRNA targets remain unknown. Our database analysis shows that YTHDF1 is highly expressed during peak cardiomyocyte differentiation and maturation in mouse embryos, with cardiac expression declining in later embryonic stages and after birth. In humans, YTHDF1 expression is higher in the left ventricle than in the right. Notably, left ventricular YTHDF1 levels are significantly reduced in patients with dilated cardiomyopathy (DCM)—a condition often linked to defective cardiomyocyte differentiation—compared to healthy controls. These results imply a role for YTHDF1 in cardiomyocyte differentiation and a potential involvement in DCM pathogenesis.

Using rat H9C2 cardiomyoblasts, we further showed that YTHDF1 knockdown attenuates cardiomyocytic differentiation. Importantly, we identified *TET2* mRNA as a direct target of YTHDF1 and demonstrated that it regulates TET2 translation during differentiation. Given that YTHDF1 binds spliceosome-associated proteins and regulates mRNA splicing [32], it may also influence *TET2* mRNA splicing during cardiac differentiation. As a key m⁶A modification reader (YTHDF1) and a DNA demethylase (TET2), both molecules represent critical epigenetic regulators. Our findings reveal a regulatory hierarchy between YTHDF1 and TET2. Complementation assays showed that the novel *tet2* variant effectively promotes cardiomyocyte differentiation in H9C2 cells. However, an important question remains: Why does the canonical variant fail to promote differentiation as efficiently? During complementation with the canonical *tet2* isoform, TET2 protein appeared at approximately 100 kDa rather than the expected ~250 kDa—a discrepancy that warrants further investigation. We speculate that these differences arise because the overexpression plasmid for the canonical sequence lacks endogenous 5’ and 3’ untranslated regions (UTRs). The absence of UTRs may facilitate YTHDF1-independent translation but could also disrupt mRNA splicing, preventing efficient production of the full-length 212 kDa TET2 protein. Of note, the TET2 antibody recognizes an epitope shared by multiple isoforms, and several bands (e.g., ~ 130 kDa and ~200 kDa) were not well resolved by SDS-PAGE. These signals may represent additional splice isoforms, proteolytic fragments, or non-specific binding. Definitive identification will require isoform-resolved approaches in future studies. The subtle differences in the amino acids encoded near the junction of exons 6 and 7 also warrant investigation. Importantly, the specific m⁶A residue(s) on *TET2* mRNA responsible for YTHDF1-mediated regulation remain unidentified. Although our current data strongly support YTHDF1’s regulation of TET2 protein levels through translational control, future studies could directly validate changes in TET2 mRNA distribution on ribosomes. Polysome profiling, for example, would provide more direct evidence of this translational regulation. Furthermore, the regulatory role of the YTHDF1-TET2 axis in the heart development requires further validation in embryonic stem cells and *in vivo*.

Generally, the proliferative and regenerative capacities of cardiomyocytes decline rapidly with age, rendering mature cardiomyocytes terminally differentiated. In adult mammals, cardiac repair after injury typically involves the replacement of functional tissue with fibrotic scarring, which significantly impairs functional recovery. In contrast, the neonatal heart exhibits a remarkable regenerative capacity, allowing complete structural and functional recovery following severe injuries such as myocardial infarction or apical resection. This study further elucidates the role of epigenetic regulation in modulating cardiomyocyte differentiation, providing valuable insights for the development of therapeutic strategies for cardiac diseases, including myocardial infarction and heart failure.

In conclusion, our study identifies *TET2* mRNA as a key functional target of the m⁶A reader YTHDF1 in regulating rat cardiomyocyte differentiation, and the YTHDF1-TET2 axis in dilated cardiomyopathy deserved further exploration.

## Supporting information

S1 File Supplementary Tables 1–3; Supplementary Figures 1–3. (DOCX)

S4 Table Supplementary Table 4, the processed RNA-Seq FPKM data. (XLSX)

S5 Table Supplementary Table 5, the processed RIP-Seq data. (XLSX)

S2 File The raw images for blots and gels. (PDF)

## Abbreviations

5hmC: 5-hydroxymethylcytosine
5mC: 5-methylcytosine
DCM: dilated cardiomyopathy
m^6^A: N6-methyladenosine
NF: Non-failing
RIP-seq: RNA immunoprecipitation sequencing
TET: ten-eleven translocation family
YTHDF: YT521-B homology domain-containing family
