# Supplementary material for: YTHDF1 impacts cardiomyocyte differentiation by regulating the TET2 mRNA
Source: PLoS One. 2026 May 15;21(5):e0349040. doi: 10.1371/journal.pone.0349040 (PMC13178915; doi:10.1371/journal.pone.0349040)
Supplement: S1 File — (DOCX) [file pone.0349040.s001.docx]

**Supplementary Tables 1-3; Supplementary Figures 1-3**

Table S1. The Sequence for YTHDF1 Knockdown.

| Name | Sequences |
| --- | --- |
| KD1 | 5’-TTCATGACAATGACTTTGA-3’ |
| KD2 | 5’-TTACCAGCACAGGTTTAATTT-3’ |
| sh-1 | 5’-CCTATGGACAACTTAGTAA-3’ |
| sh-2 | 5’-GGATGTGCCCAATAATCAA-3’ |
| sh-3 | 5’-GCACACAACCTCCATCTTT-3’ |

Table S2. The Primers for Q-PCR.

| Primer name | Sequences |
| --- | --- |
| ythdf1-F | 5’-TCTGCTCTTCAGTGTCAATGG-3’ |
| ythdf1-R | 5’-GATGGAGGTTGTGTGCTTATAGG-3’ |
| myog-F | 5’-GTCCCAACCCAGGAGATCATT-3’ |
| myog-R | 5’-AGCCTGGCAGACAATCTCAG-3’ |
| myod-F | 5’-CTGCTCTGATGGCATGATGGA-3’ |
| myod-R | 5’-CACTATGCTGGACAGGCAGT-3’ |
| mef2c-F | 5’-ATGGATGAACGTAACAGACAGG-3’ |
| mef2c-R | 5’-ATGGATGAACGTAACAGACAGG-3’ |
| tnnt2-F | 5’-CAGGAGGAAGGCTGAAGATG-3’ |
| tnnt2-R | 5’-CGGCCTCTAGGTTGTGGATA-3’ |
| ttn-F | 5’-AGGAACTCCTCCTCCCCATC-3’ |
| ttn-R | 5’-CTCGTGCTGGTTTCTTCCCT-3’ |
| gapdh-F | 5’-CAAGATTGTCAGCAATGCATCC-3’ |
| gapdh-R | 5’-AGTTGCTGTTGAAGTCACAGG-3’ |
| tet2-F | 5’-GTCGAGTTTGAACACCGAGC-3’ |
| tet2-R | 5’-GTGACCACCACTGTACTGCC-3’ |
| actb-F | 5’-CACACAGAGTACTTGCGCTC-3’ |
| actb-R | 5’-CACCATGTACCCAGGCATTG-3’ |
| tet2-truncated exon3-F | 5’-GGAAACCCAGAAAAAGATGAAG-3’ |
| tet2-truncated exon3-R | 5’-AGCTCTTGCCTTCTTTACCAG-3’ |
| tet2-exon3-F | 5’-CAGGTGCTTTCAAGAACAGG-3’ |
| tet2-exon3-R | 5’-ATCCAGGCTCAATCTTGATTGG-3’ |
| tet2-exon10-F | 5’-AAAGCTAGAAAGCTTTCTTCTCTGG-3’ |
| tet2-exon11-R | 5’-GGCATCTCCGTACATATCTG-3’ |

Table S3.The antibodies for Western Blot.

| Antibodis | Source |
| --- | --- |
| Anti-YTHDF1 | Proteintech; Cat No. 17479-1-AP |
| Anti-MYOG | Ab-mart; Cat No. T58830 |
| Anti-TET2 | Beyotime; Cat No. AF8119 |
| Anti-cTnT | Proteintech; Cat No. 68300-1-Ig |
| Anti-cTnI | Proteintech; Cat No. 66376-1-Ig |
| Anti-GAPDH | Proteintech; Cat No. 60004-1-Ig |
| Anti-5mC | Proteintech; Cat No. 68301-1-Ig |
| Anti-5hmC | Active Motif; Cat No. 39069 |
| HRP-labeled Goat Anti-Rabbit IgG (H+L) | Beyotime; Cat No. A0208 |
| HRP-labeled Goat Anti-Mouse IgG (H+L) | Beyotime; Cat No. A0216 |

**Figure S1. YTHDF1 knockdown validation and its effects on TET2 expression. a.** Quantification of Figure 1e. **b, c.** YTHDF1 expression levels following knockdown by three shRNAs targeting rat YTHDF1. One-way ANOVA was used (n=3). **d, e.** TET2 protein levels during cardiomyocyte differentiation in control and sh-1 (high-efficiency) YTHDF1-knockdown H9C2 cells: 10% SDS-PAGE (d); 7.5% SDS-PAGE (e). **f, g.** Quantification of 5mC and 5hmC levels in sh-NC and sh-YTHDF1 H9C2 cells. Unpaired two-tailed t-test was used (n=8/ n=9). **h.** Transcript levels of tet1, tet2, and tet3 in H9C2 cells from RNA-Seq data.


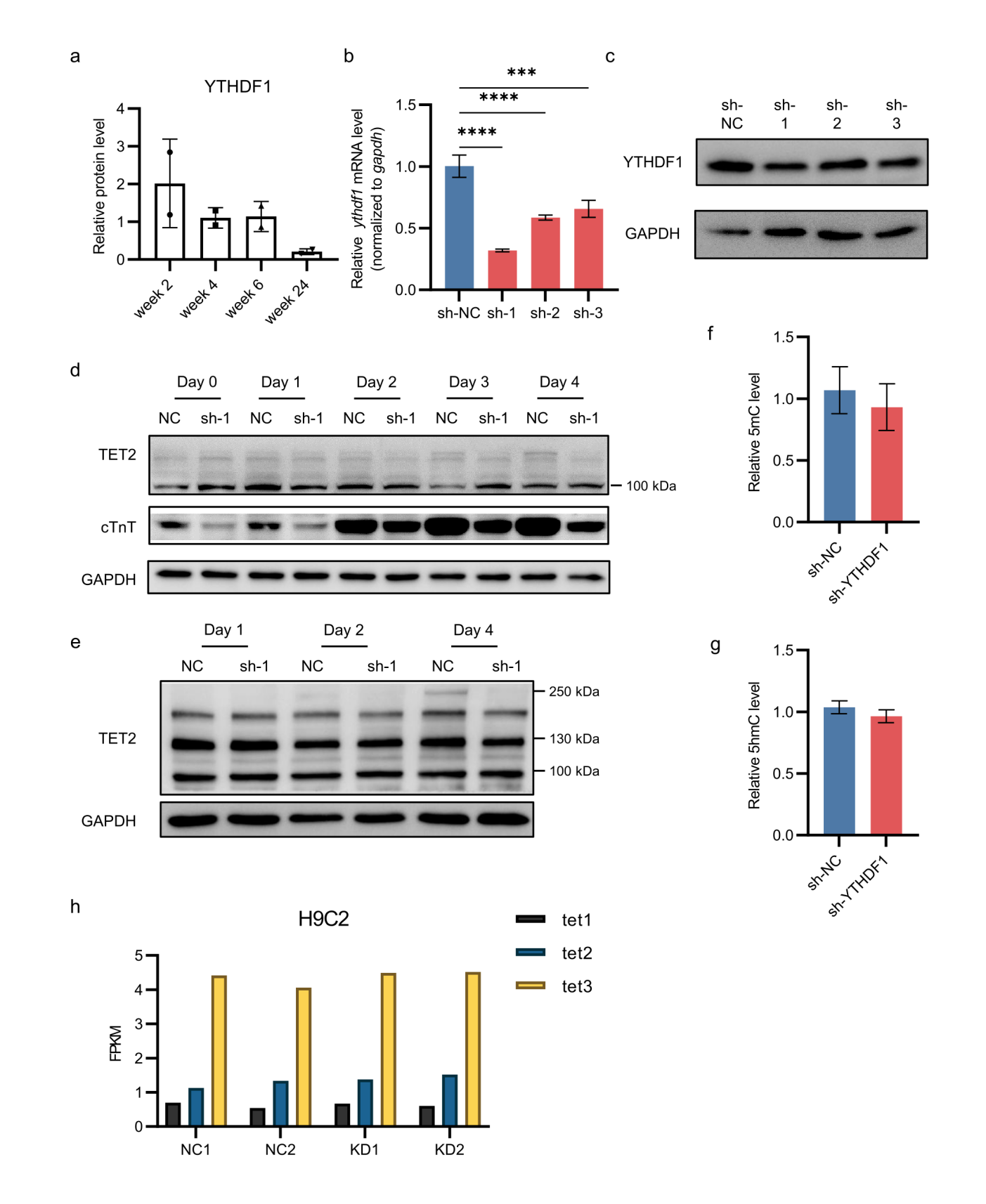


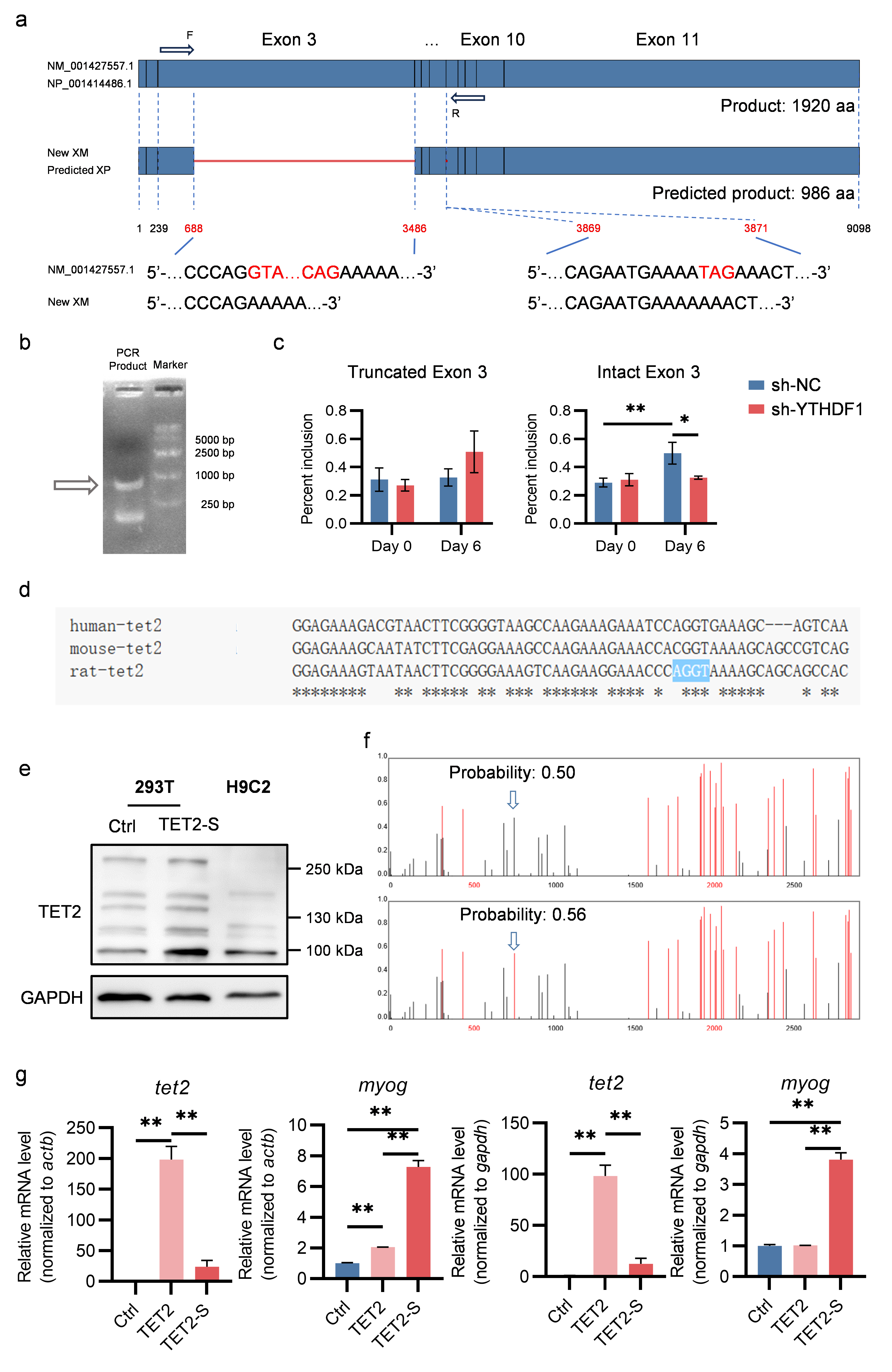


**Figure S2. Alternative splicing of rat *TET2* mRNA and expression analysis of variants.** **a.** Schematic of the two *TET2* mRNA variants. Red lines and bases indicate exon regions absent in the canonical reference. **b.** About 1000 bp PCR product amplified with primers F and R (showed in Figure S2a) in H9C2 cells. **c.** Exon 3 inclusion level before and after differentiation. Truncated Exon 3, the new XM of tet2; intact exon 3, the *TET2* mRNA with relatively intact exon 3. Two-way ANOVA was used (n=3). **d.** Cross-species comparison of the rat *tet2* exon 3 splice-junction region across human, mouse, and rat. **e.** TET2 protein levels following transient transfection of the novel *tet2* variant (TET-S) in 293T cells. **f.** m⁶A site prediction for new XM (upper) and *TET2* NM_001427557.1 with alternative exon 3 as new XM (lower). Red line indicates high-confidence sites. Blue arrows indicate the divergent exon 7 splice sites in both variants. **g.** *tet2* and *myog* transcription levels relative to *gapdh* or *actb* at day 5 of cardiomyocyte differentiation in sh‑1 (high‑efficiency) YTHDF1‑knockdown H9C2 cells with transient complementation of *tet2* variants. One-way ANOVA was used (n=3).


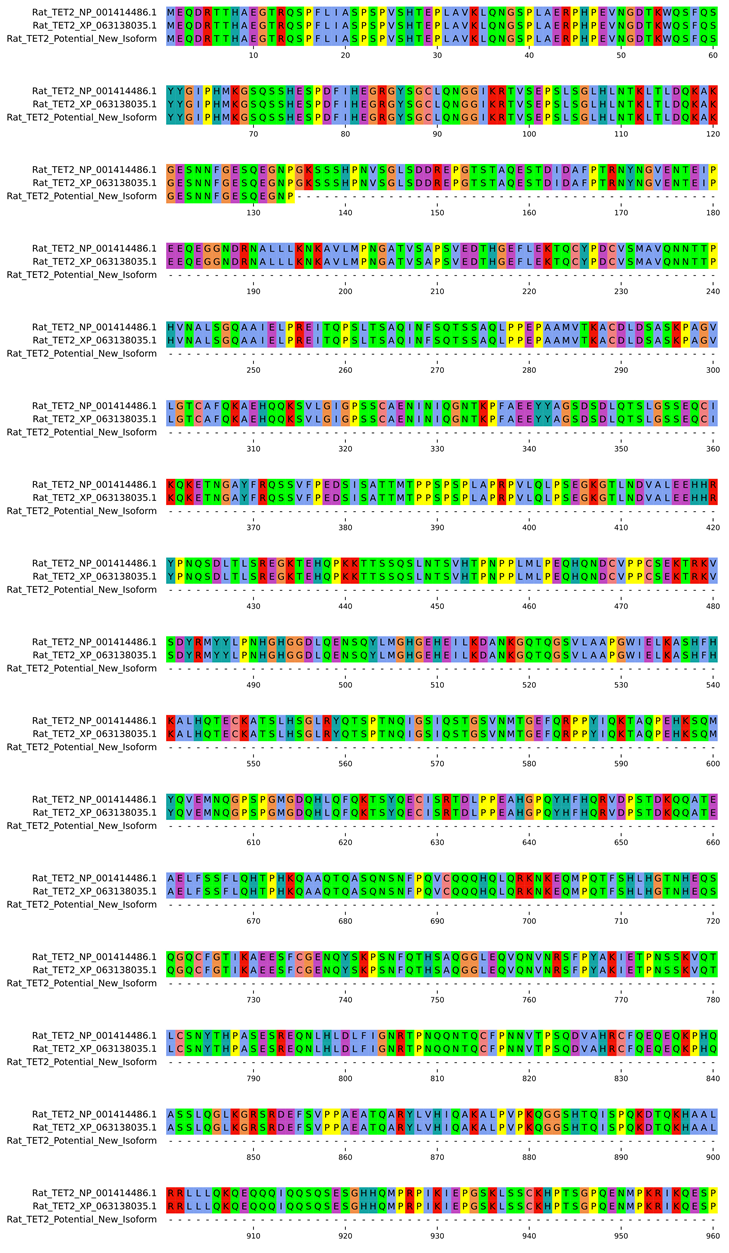


**
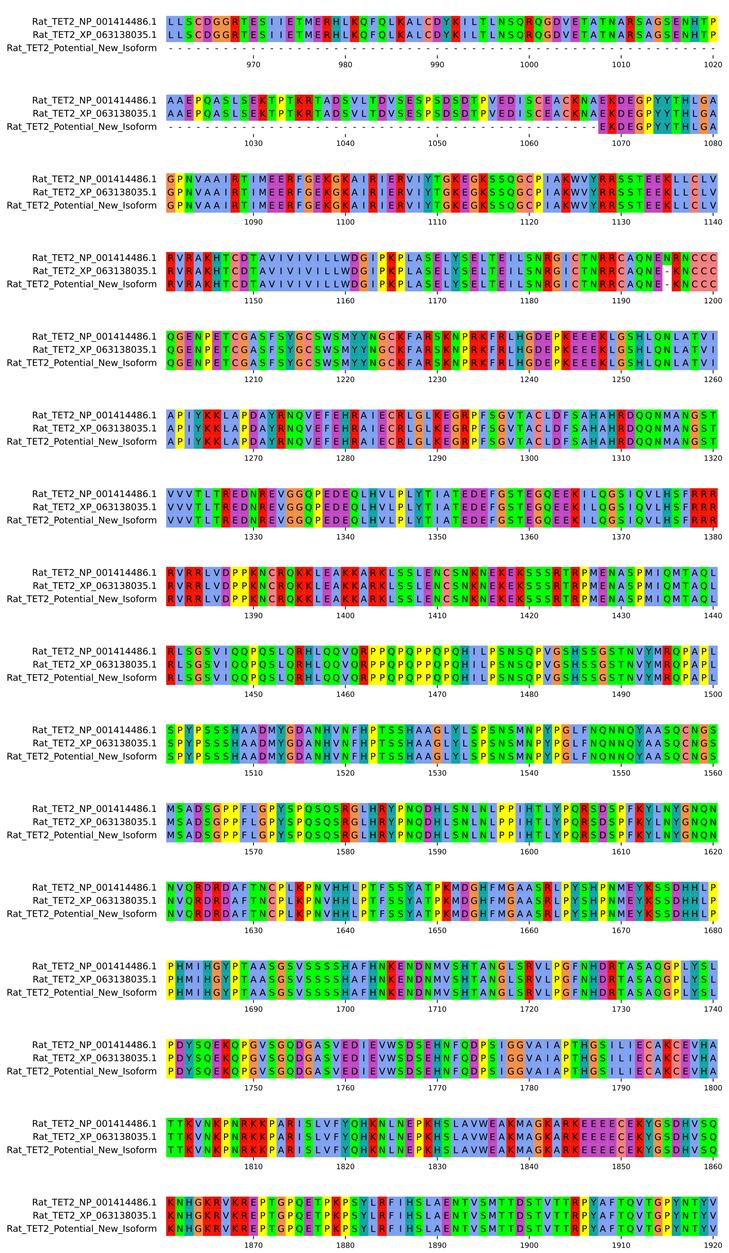
**

**Figure S3.** **Amino acid sequence alignment of the three rat *tet2* variants.**
