# Supplementary material for: YTHDF1 impacts cardiomyocyte differentiation by regulating the TET2 mRNA
Source: PLoS One. 2026 May 15;21(5):e0349040. doi: 10.1371/journal.pone.0349040 (PMC13178915; doi:10.1371/journal.pone.0349040)
Supplement: S2 File — (PDF) [file pone.0349040.s004.pdf]

Figure 1e

40 kDa
30 kDa

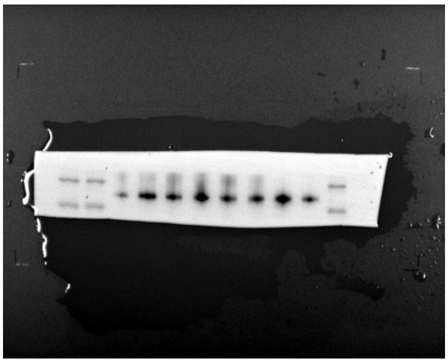

GAPDH

70 kDa
55 kDa

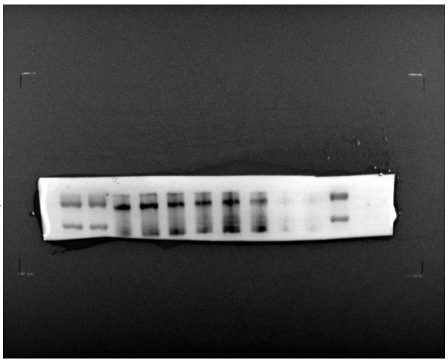

YTHDF1

Figure 2a

70 kDa
55 kDa

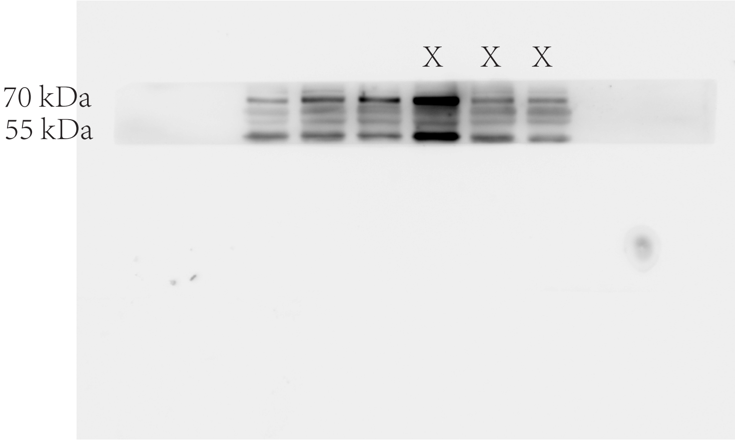

YTHDF1

40 kDa

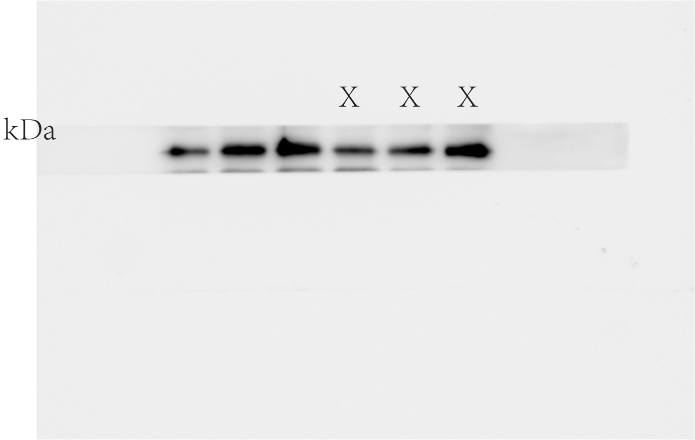

MYOG

40 kDa

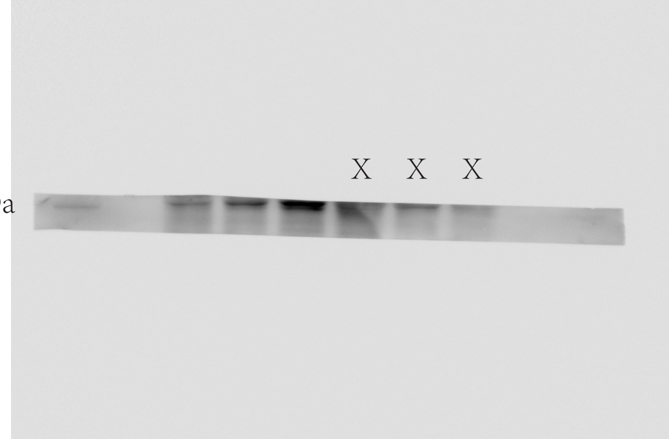

cTnT

35 kDa

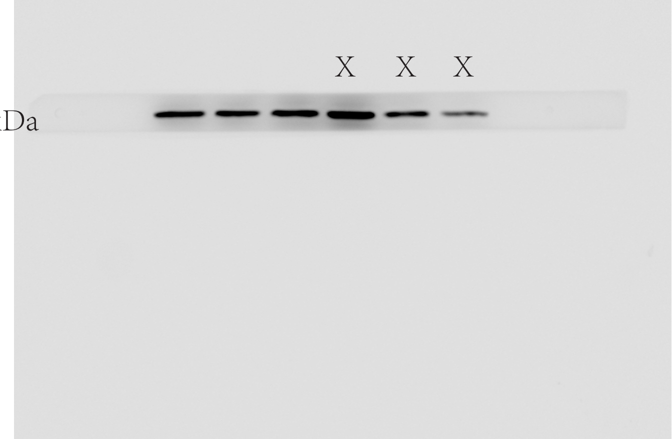

GAPDH

Figure 2c

70 kDa
55 kDa

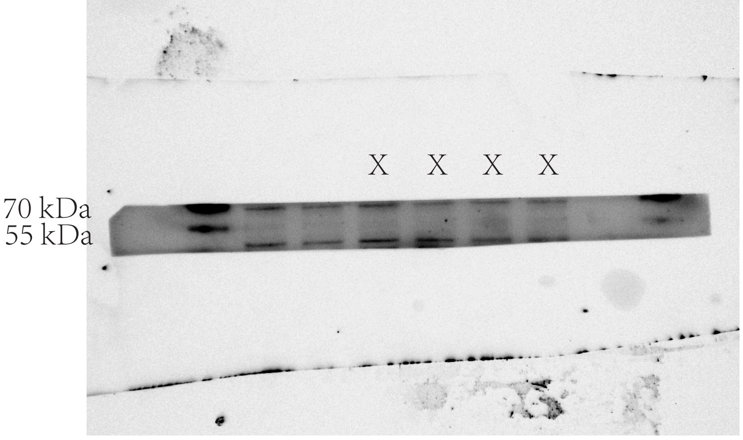

YTHDF1

35 kDa

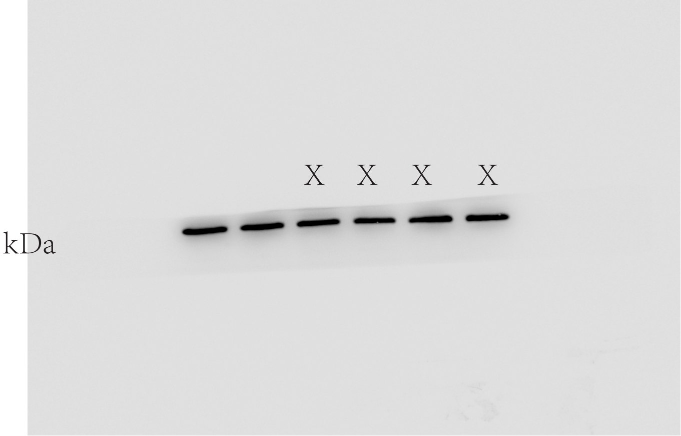

GAPDH

Figure 2g

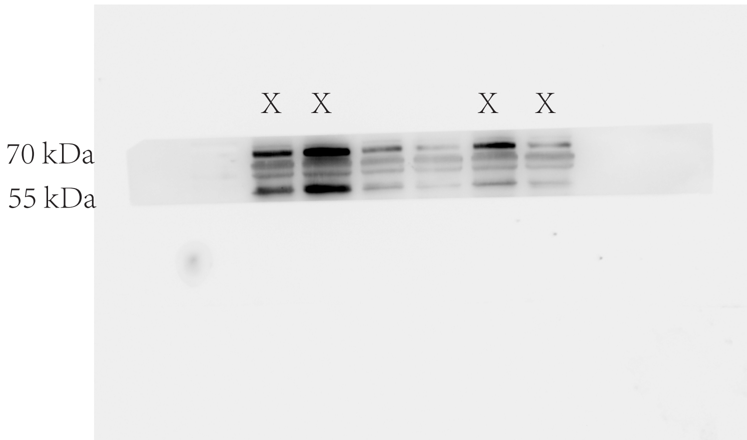

YTHDF1

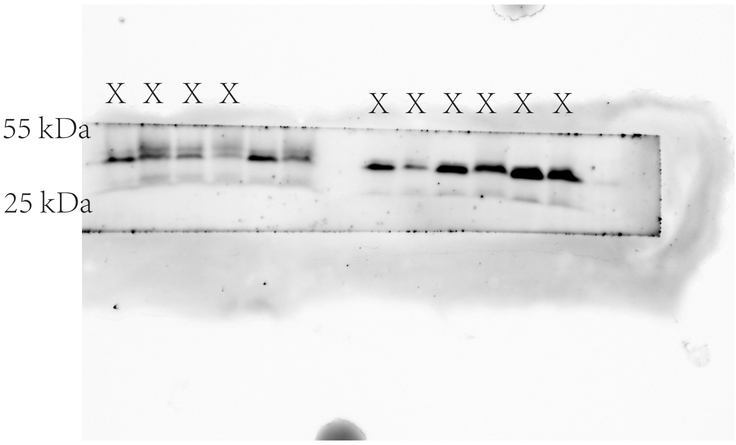

MYOG

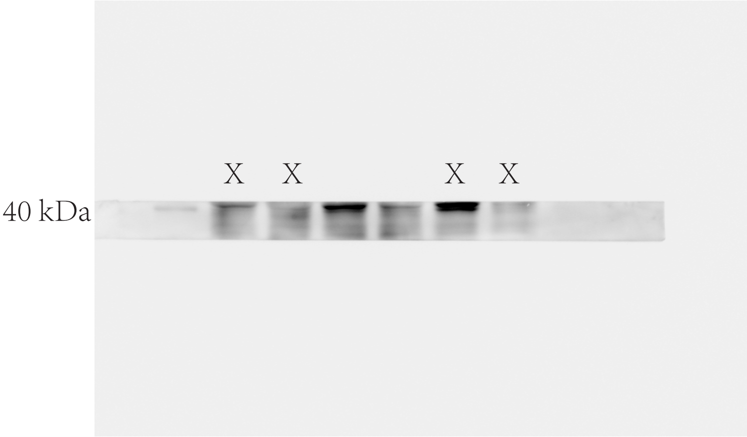

cTnT

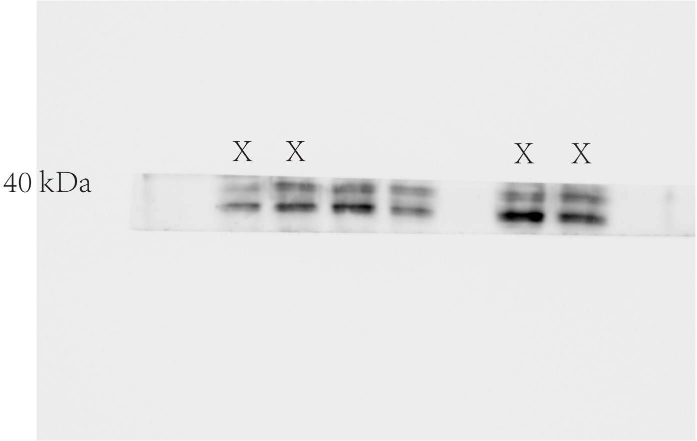

cTnI

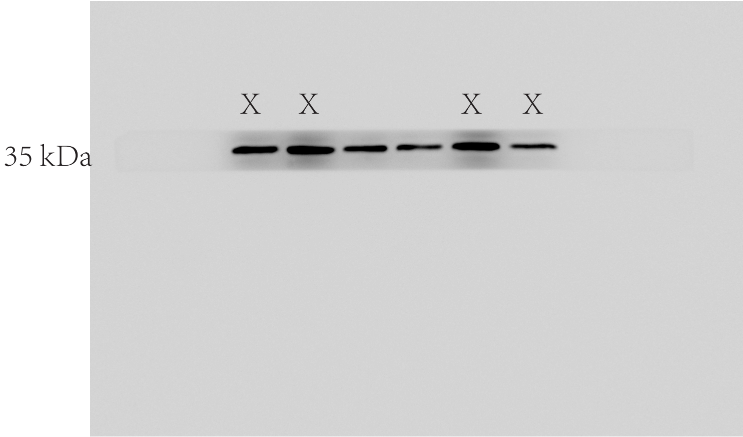

GAPDH

Figure 4c

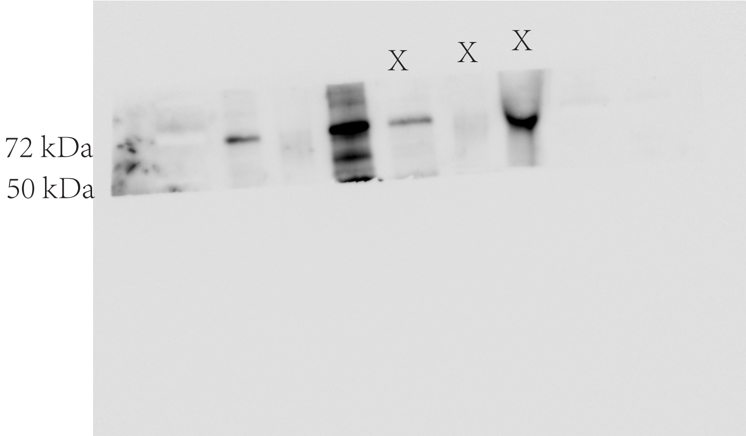

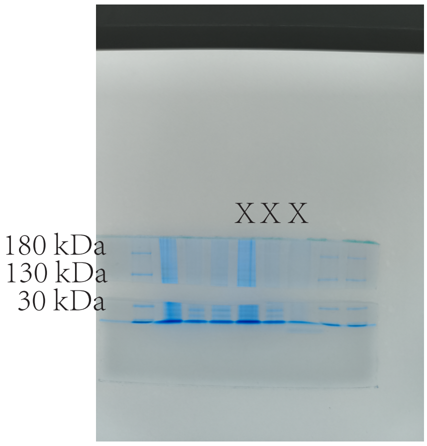

Figure 4e

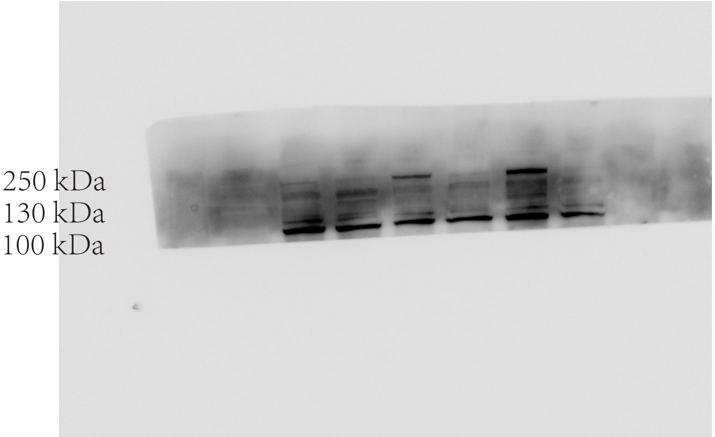

TET2

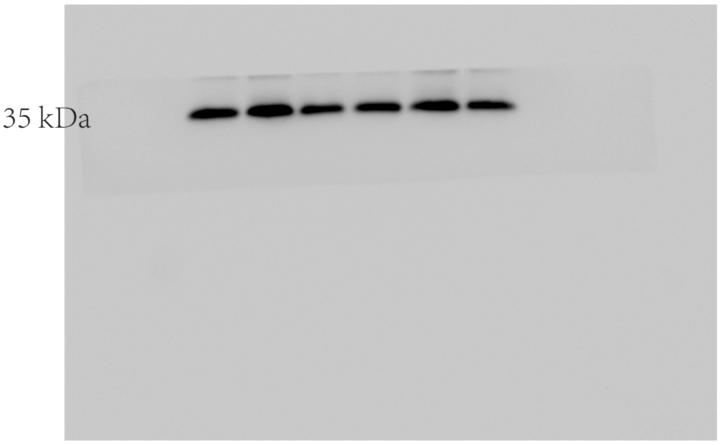

GAPDH

Figure 4f

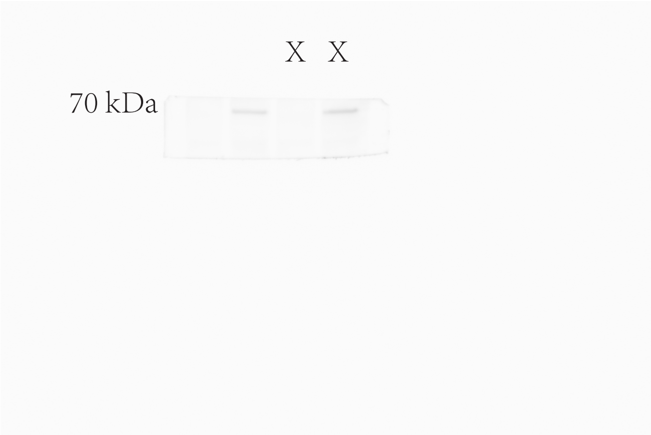

Flag

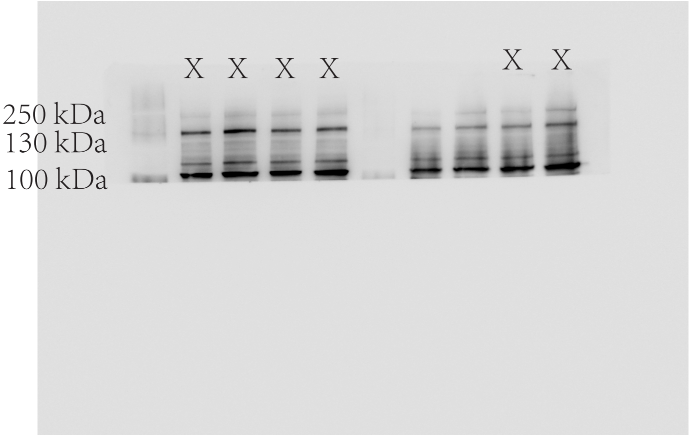

TET2

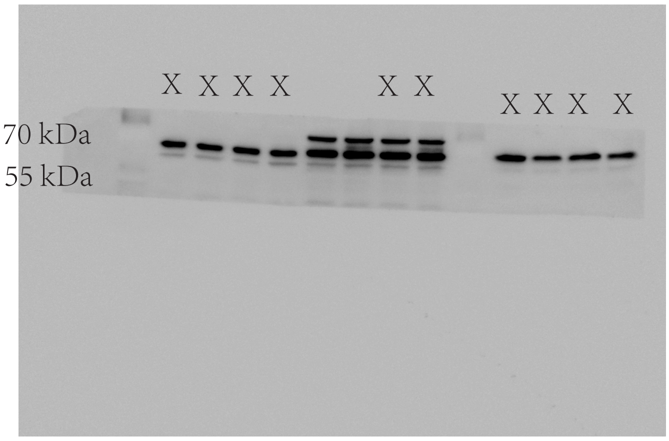

YTHDF1

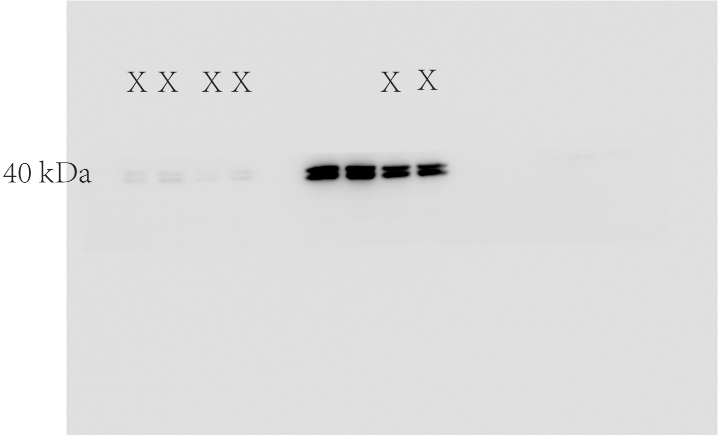

cTnT

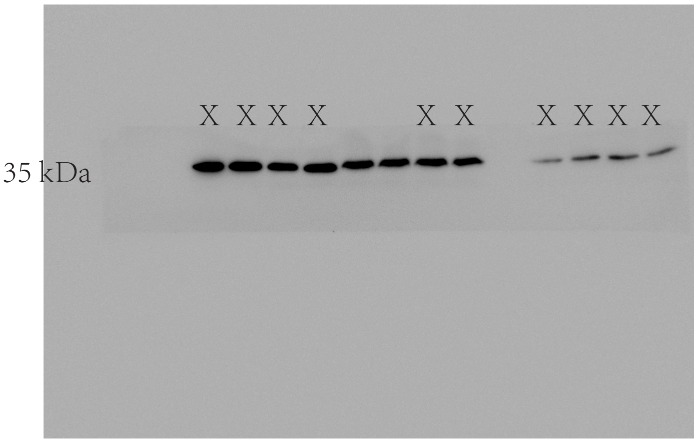

GAPDH

Figure 4g

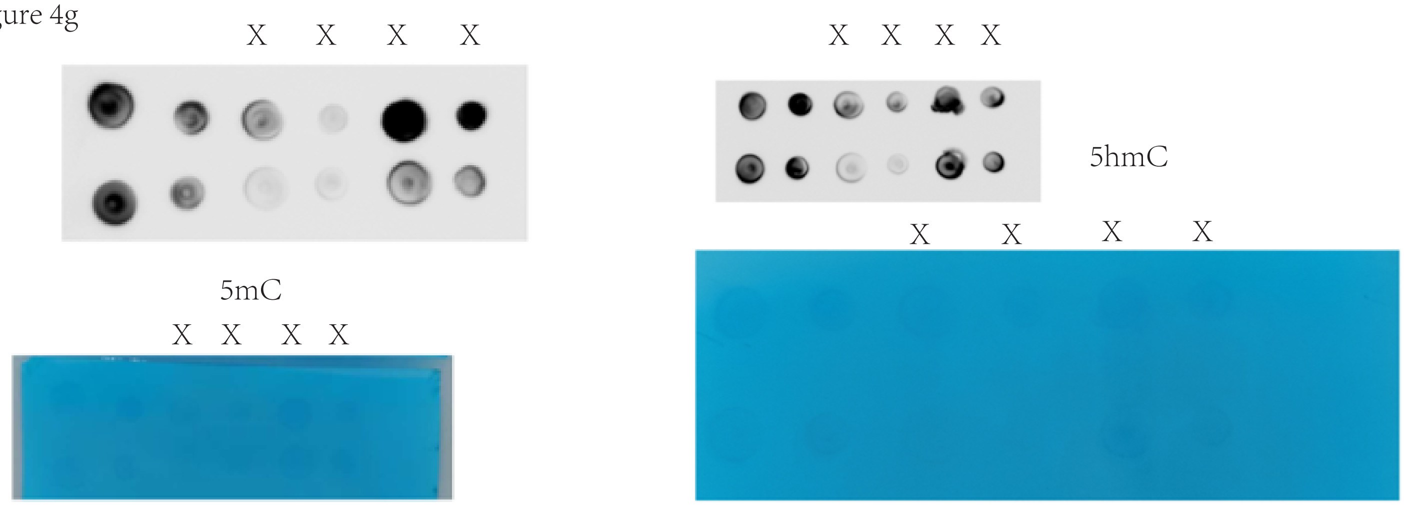

Figure 5c

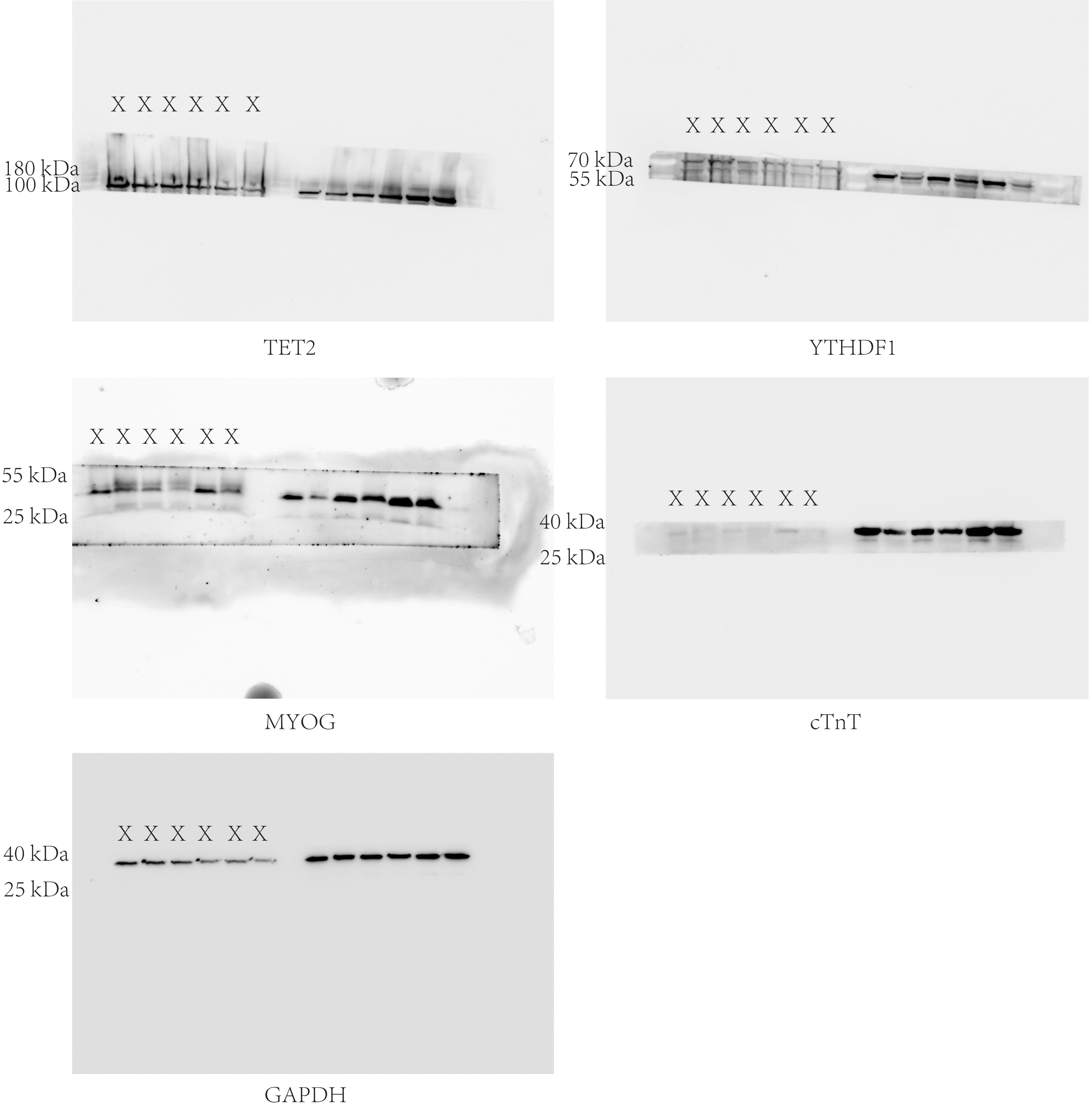

Figure S1c

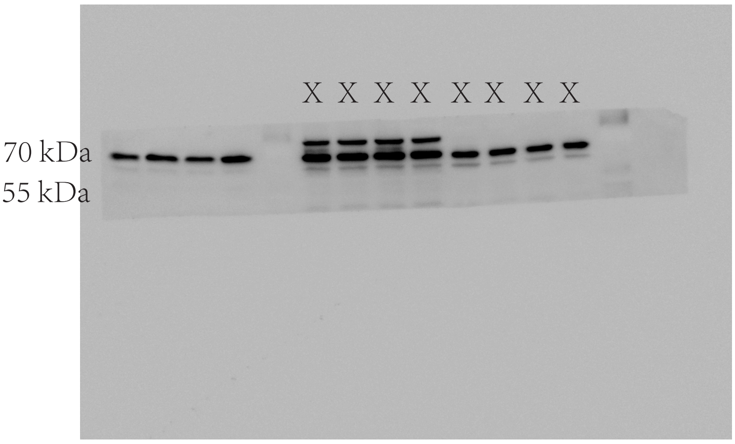

YTHDF1

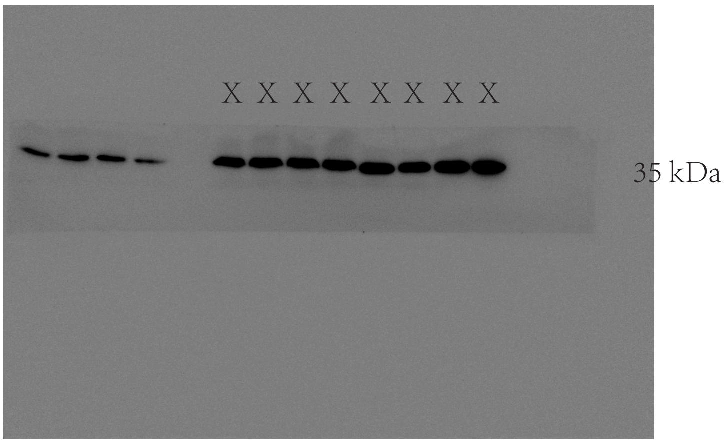

GAPDH

Figure S1d

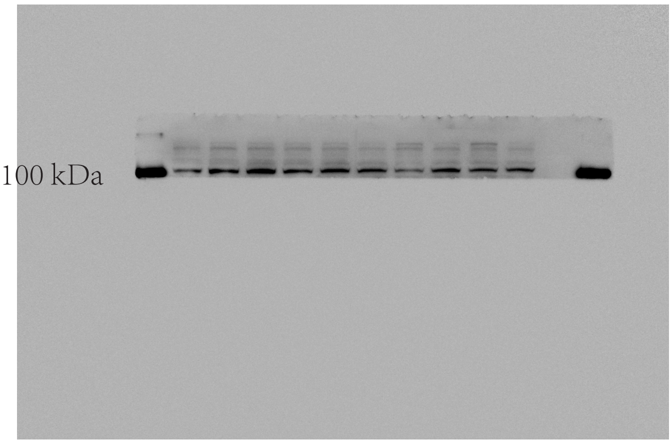

TET2

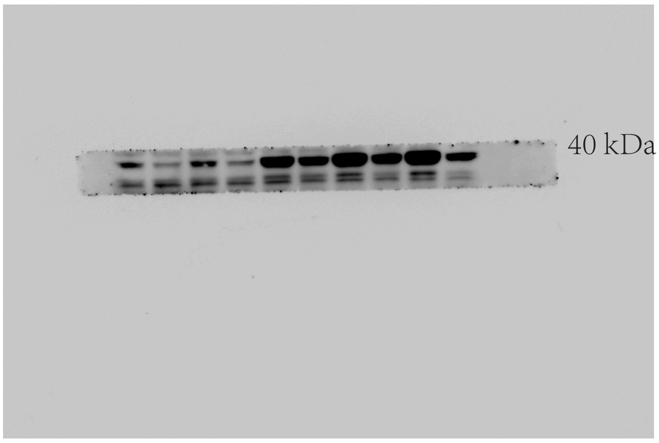

cTnT

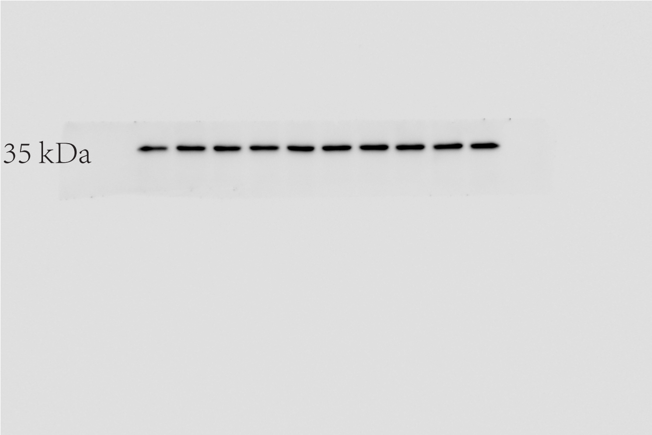

GAPDH

Figure S1e

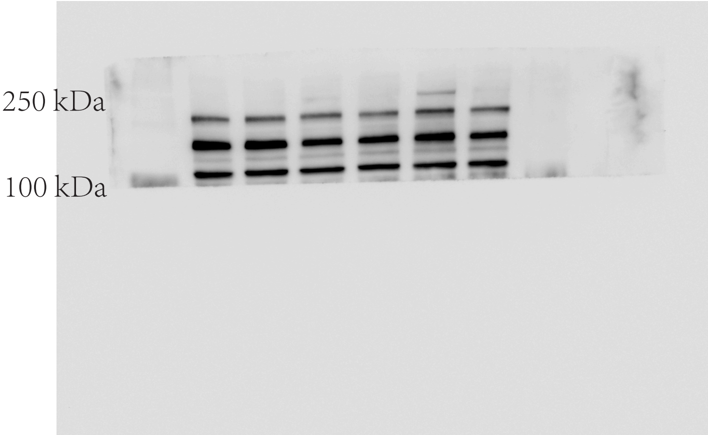

TET2

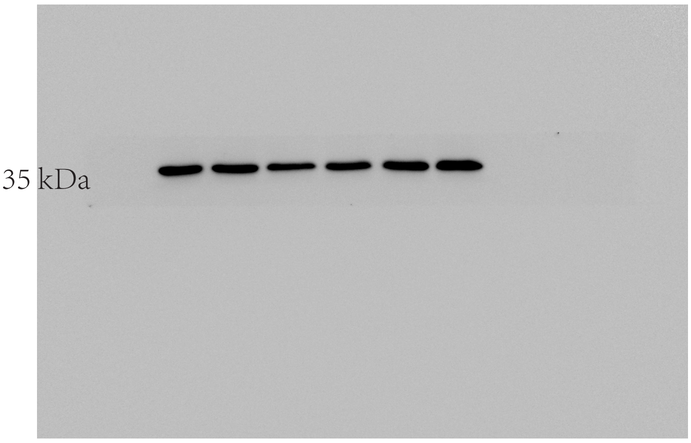

GAPDH

Figure S2b

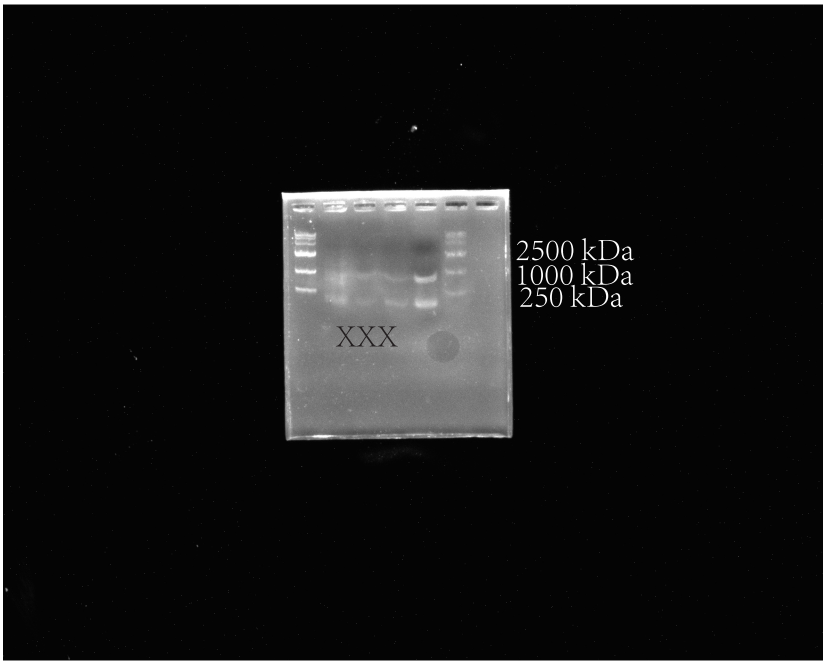

Figure S2e

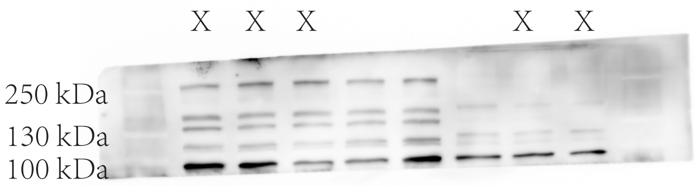

TET2

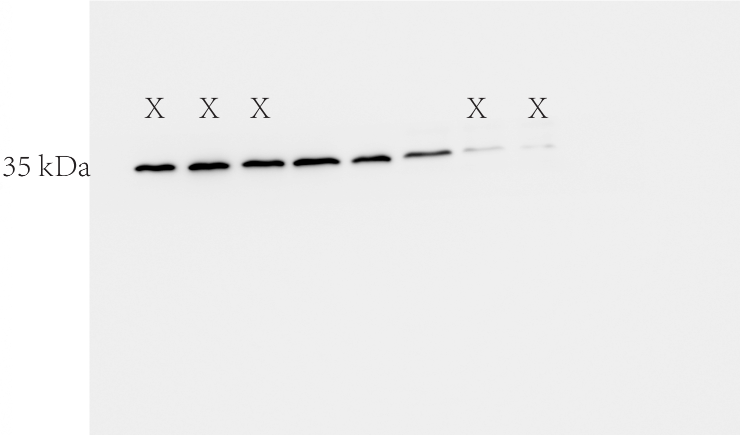

GAPDH
